# Three-dimensional patterns from the thin-film drying of amino acid solutions

**DOI:** 10.1038/srep10926

**Published:** 2015-06-03

**Authors:** Xuehua Zhang, Alexandru Crivoi, Fei Duan

**Affiliations:** 1 School of Civil, Environmental and Chemical Engineering, RMIT University, Melbourne 3001, Australia; 2 School of Mechanical and Aerospace Engineering, Nanyang Technological University, 639798, Singapore

## Abstract

Experimental atomic force microscopy (AFM) images show the dried-in patterns from amino acid solutions which can be in the form of dots or networks. The three-dimensional lattice-gas Kinetic Monte Carlo (KMC) model is applied to simulate the formation of dot-like and network-like particle structures from the evaporating thin films of solutions. A sigmoidal jump in the chemical potential value is implemented to obtain dual-scale structures with the grain size distribution peaking at two distinctive values. The simulated and experimental results are qualitatively comparable.

Particle self-assembly from evaporation attracts intense attention since the complexity of the drying process is related to multiple properties of solutes and solvents^1^,^2^,^3^. Deegan *et al.* showed the coffee-ring effect forming in the drying sessile droplets^4^. Yunker *et al.* stated that the ellipsoidal particle shape led to the uniform deposit while the spherical particles are driven by the capillary flow to form coffee rings^5^. The surfactant addition to colloidal fluids was found to reduce the suspended particle attraction and enhance the ring formation in the drying patterns of the solutions^6^, while the surfactant-induced Marangoni eddy flows suppressed the coffee-ring effect in the other experiments^7^. Grzelczak *et al.* summarized that the directed nanoparticle self-assembly could be guided both by the intrinsic characteristics of nanoparticles including particle species, particle shape, or solvent polarity, and the external surrounding properties including electric fields, magnetic fields, or macroscopic flow field^8^. The wettability and drying regime of liquids can be modified not only by altering the particles and base fluids^9^, but also by modifying the surface of substrates^10^. Highly ordered spherical colloidal supraballs were created on a superhydrophobic surface by controlling evaporation and particle diffusivity^11^. Evaporation-induced self-assembly of chemically converted graphene into a monolayer was achieved on a hydrophobic substrate and significantly changed the surface properties^12^,^13^. The bowl-shaped deposition was inverted from drying the water suspension containing the alumina powder on the hydrophobic surface of wax-treated fibrous copper oxide substrates. For biological applications, the homogeneous protein shell with an internal vacuole was made from a pendant protein droplet under a hydrophobic surface^14^, while the lysozyme solution evaporation on the superhydrophobic patterned poly(methyl methacrylate) surfaces resulted in the hollow spherical residues^15^. Gentile *et al.* further fabricated the superhydrophobic surface for the evaporative patterning of the diluted biological solutions^16^. They demonstrated the multi-layered micropillars could trap the molecules in a low molar range to aggregate for the accurate detection. The drying amino acid solutions, in which amino acids are diluted in water or the other solvents, can be found in food processing^17^, pharmaceutical industry^18^,^19^, biomedical diagnosis^20^,^21^, and the others. Amino acids could be extracted as the solvents were evaporated^17^,^18^,^19^,^20^,^21^. However, the study of the pattern formation from evaporating thin films of amino acid solutions is very limited, although the patterns emerging from the drying thin film of the nanoparticle suspensions have been studied in numerous theoretical and experimental works^22^,^23^,^24^,^25^. Among many others, the reported results included the formation of nanorings^26^, three-dimensional (3D) stalagmites^27^ and dual-scaled granular and fingering structures^28^. The formation of dual-scaled structures has been investigated in a two-dimensional (2D) domain using a pseudo-3D model^24^,^28^, and obtaining complex structures in 3D relied on simulating a non-uniform diblock copolymer substrate^23^. Here we introduce a combined approach using a full 3D domain for a thin film representation, and applying a “sigmoidal” jump in the effective chemical potential values to simulate the formation of dual-scaled networks in three dimensions. The simulated results are compared with the observation of the dried patterns in the thin film of the amino acid solutions under an atomic force microscopy (AFM).

## Results

The selected amino acids, L-Lysine monohydrocholoride (LysHCl), glutamic acid (Glu) and glycine (Gly), were prepared into the solutions, then the solutions formed thin films on the mica substrate and dried naturally for the measurement. The representative AFM images can be seen in three types of particle self-assembled morphologies: flat disks, stripe-like islands and cellular networks from the dried amino acid solutions. The experimental results are compared with the following simulation.

**Figure 1:**
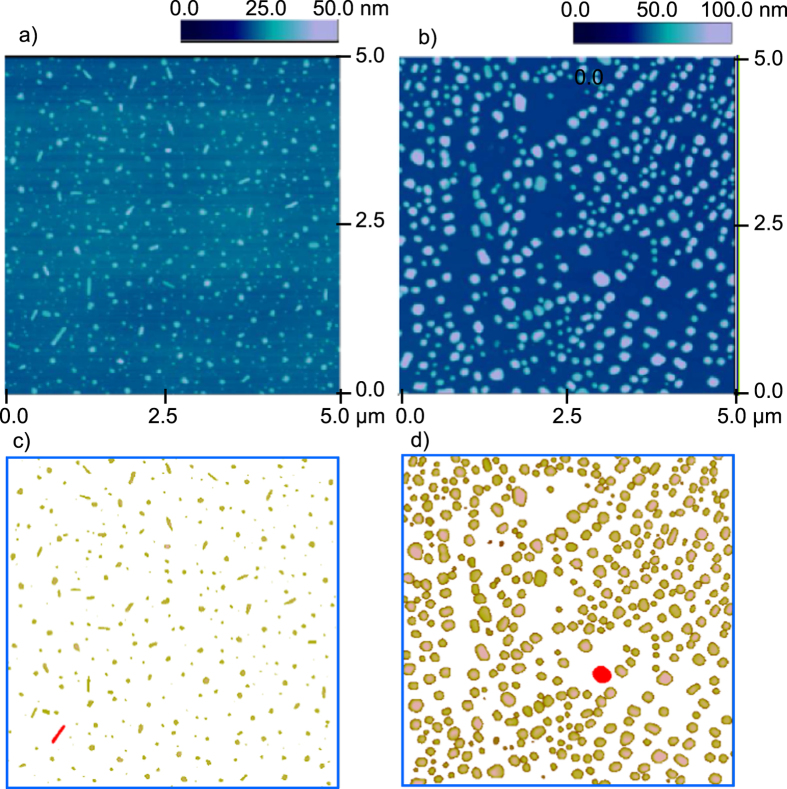
AFM height images and the corresponding images only showing the grains. (**a**) From evaporation of 5 mM LysHCl solution. (**b**) From evaporation of 10 mM Gly solution. An example grain is labeled by red in each image in (**c**) and (**d**).

**Figure 2:**
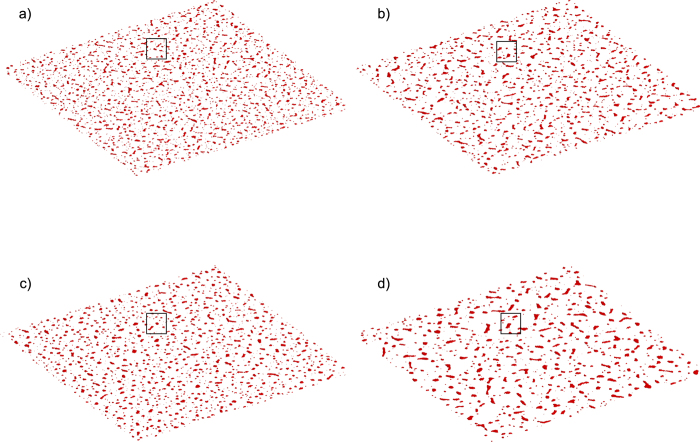
Simulation results showing the dotted patterns. (**a**) *N*_*mov*_ = 5, *μ* = − 3.7, *kT* = 0.2. (**b**) *N*_*mov*_ = 20, *μ* = − 3.7, *kT* = 0.2. (**c**) *N*_*mov*_ = 20, *μ* = − 3.7, *kT* = 0.25. (**d**) *N*_*mov*_ = 20, *μ* = − 3.6, *kT* = 0.2. Other parameters: *ϕ* = 0.5%, *ε*_*nn*_ = 2, *D* = 400, *z* = 0.05. The particle aggregation is selected in the square shown in the figure to display the trend of change in the parameter study.

Figure 1 illustrates the experimental results for the dried solutions of the amino acids, LysHCl and Gly. The morphologies of LysHCl particles have a “dotted” pattern composed from small disks of various diameters and short stripe-like islands in Fig. 1. The similar patterns have been reported to form from the CdSe and PbSe nanocrystals^22^, and thus may be considered as a universal class of patterns. The analysis of grain sizes in LysHCl patterns shows that the number of grains in the area of 25 *μ*m^2^ is 352, and the minimal grain size is 95 nm^2^ and the maximal, 14877 nm^2^ with all the particles less than 4 nm filtered out. The mean grain size is 2351 nm^2^, and the standard deviation is 2183 nm^2^. The length of the stripe-like islands formed by LysHCl varies from several nanometers up to 350 nm, but the width is quite uniform around 100 nm. The same analysis of grain sizes in Gly patterns shows that the number of grains is 363, which is much higher than the grain density of LysHCl pattern. The minimal grain size in Gly pattern is 762 nm^2^ and the maximal 84018 nm^2^. The mean grain size is 18740 nm^2^ while the standard deviation is 12353 nm^2^. Hence the dots formed by Gly are larger than those by LysHCl. The simulations produce the final dotted patterns, presented in Fig. 2.

The parameter study indicates that the small islands of various size, shape and density can be formed in the end of the simulation runs with the particle volumetric concentration at *ϕ* = 0.5% in the whole simulation domain. The specific characteristics depend on the other parameter values. Slightly increasing *kT* from 0.2 to 0.25 leads to the emergence of slightly smaller and rounder dots in the structure (see Fig. 2(b,c)). At the same time decreasing particle mobility, *N*_*mov*_, leads to smaller and more uniformly packed dots (compare Fig. 1(a,b)). Changing the chemical potential value from –3.7 in Fig. 2(b) to –3.6 in Fig. 2(d) leads to a smaller number of larger dots of a similar shape statistically in the whole simulation domain. The similar pattern behaviors have been observed in 2D simulation results as well^22^,^28^. To enhance the statement, a square region is selected to emphasize the feature in Fig. 2. The trends in the 3D simulation results presented in the current work are compatible with the original 2D simulations.

For the simulation of more complex dual-scale structures, the idea previously used by Stannard *et al.* and Pauliac-Vaujour *et al.*^24^,^28^ in their “pseudo-3D” model is implemented in the present fully-3D model. It is assumed that the effective chemical potential value, *μ*, is dependent on the global liquid coverage of the substrate. As the percentage of the fully dried substrate reaches a certain critical value, the chemical potential value experiences a sharp “sigmoidal” jump leading to a very rapid dry-out of the rest of the remaining solvent. Analytically, the described process is presented by the following equation^28^,
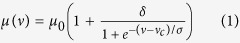
where *μ*_0_ is the initial chemical potential value, *δ* and *σ* are the parameters indicating the width and the sharpness of the sigmoidal jump respectively, *ν* is the current fraction of the dried substrate, and *ν*_*c*_ is the critical coverage value.

**Figure 3:**
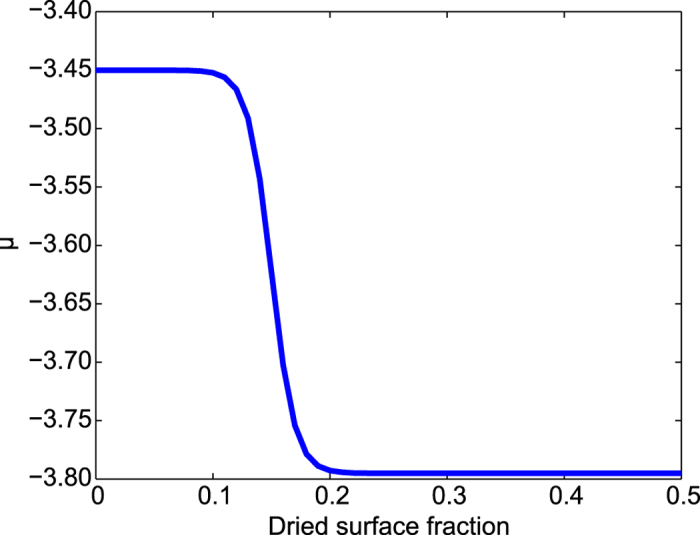
Example of a sigmoidal jump function for the chemical potential value. Parameters: *μ*_0_ = − 3.45, *ν*_*c*_ = 0.15, *δ* = 0.15, *σ* = 0.01.

Figure 3 shows a graphical example of the sharp change in the *μ* value when the percentage of the dried surface approaches a critical value of 15%. The assumed dependence on the global substrate coverage is attributed to the presence of the disjoining pressure factor in the evaporation of a thin film. It uses the assumption that the non-dimensional parameter of *μ* includes both “true” solvent chemical potential and the disjoining pressure influence in its “effective” value which is used in the Monte Carlo algorithm. The similar approach has been previously used to achieve dual-scale network-like and fingering structures on a 2D lattice domain and showed a good agreement with the experimentally observed nanoparticle structures^24^,^28^.

**Figure 4:**
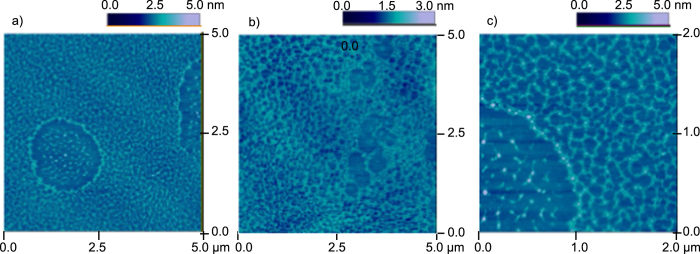
AFM images showing cellular patterns from drying 5 mM Glu solution.

Remarkably, the similar dual-scale network-like structures have been observed in our experiments with the amino acid solutions as well. Figure 4 shows the experimental results from drying the solutions of Glu. The results show that different combinations in the network grains distributions are possible. The length of the each cellular rim ranges from 100 nm to 200 nm, and the height of the rim is around 0.5 nm. The diameter of the large circle in the center of Fig. 4(a) is 1.8 *μ*m.

**Figure 5:**
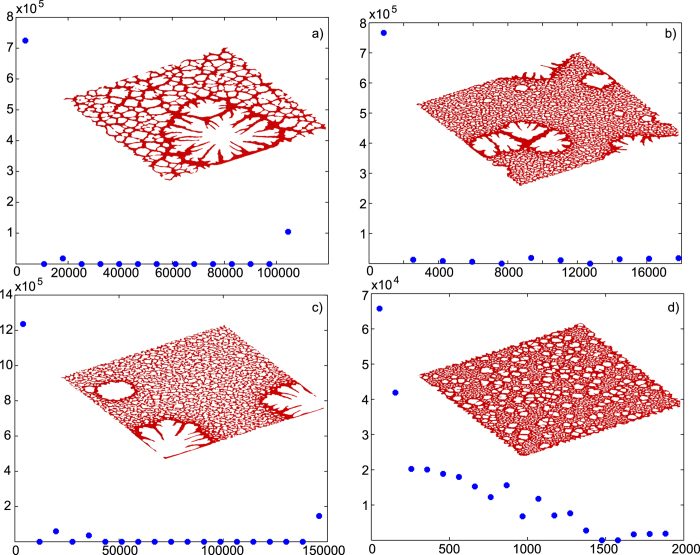
Simulation results showing the cellular patterns. (**a**) *ϕ* = 2%, *N*_*mov*_ = 30, *μ*_0_ = − 3.45, *kT* = 0.17, *δ* = 0.05. (**b**) *ϕ* = 2.5%, *N*_*mov*_ = 20, *μ*_0_ = − 3.45, *kT* = 0.2, *δ* = 0.15. (**c**) *ϕ* = 2%, *N*_*mov*_ = 50, *μ*_0_ = − 3.45, *kT* = 0.17, *δ* = 0.15. (**d**) *ϕ* = 3%, *N*_*mov*_ = 20, *μ*_0_ = − 3.6, *kT* = 0.2, *δ* = 0.15. The other parameters: *D* = 800, *z* = 0.025, *ε*_*nn*_ = 2, *ν*_*c*_ = 0.15. The plots under the images indicate the corresponding granular size distribution. The values on the horizontal axes show the size range of the connected holes in the pattern; the vertical axes stand for the total area covered by all the holes in the corresponding size range.

The simulated results in Fig. 5 show the influence of the various parameters on the resulted cellular patterns and their corresponding granular size. A smaller value of *kT* results in a smaller number of large primary grains with the possible additional fingering structures inside them (Fig. 5(a,c); by “primary” we assume those grains which start to grow before the jump in the *μ* value). Increasing *kT* leads to a higher number of primary cells (see Fig. 5(b)) and the additional change of *μ*_0_ from –3.45 to –3.6 leads to a sharp increase in a number of primary grains, which become much smaller in the case, as shown in Fig. 5(d). Additionally, the smaller value of *δ* leads to emergence of larger secondary grains (Fig. 5(a)), while increasing the value of *δ* results in uniform network-like structures of secondary grains (Fig. 5(b–d)). The observed trends well agree with the similar parameter studies, previously performed for the “pseudo-3D” model^28^. An additional analysis of the grain area distribution has been performed as well. The data shows that the grains at two separate length scales contribute to the overall coverage generally. The results are still not very clear, since (i) smaller grains have a significantly larger total area; (ii) the stochastic nature of the process produces a broad range of grain sizes; and (iii) some grains coalesce together and are captured as one larger grain. However, the gap between the two characteristic ranges is usually present, as shown in the insets of Fig. 5(a,c).

The overall distribution in the present work is qualitatively different from the pore size distribution in the fractal-like porous structure analyzed by Gentile *et al.*, where the total count of pores decays exponentially with size, and thus the total coverage does not have any local peaks at larger length scales^29^.

## Discussion

The thin-film dried patterns from the LysHCl, Glu, or Gly water-based solutions are in the form of flat disks, stripe-like islands or cellular networks on the mica substrates. The 3D lattice-gas Kinetic Monte Carlo (KMC) model is developed to simulate the formation of particle structures after the water is fully evaporated from the solutions. The sigmoidal jump in the chemical potential value is implemented to obtain the dual-scale structures with the grain size distribution characterised by the two distinctive length scales.

The trends in the simulation results show a rather interesting correspondence to the experimentally observed patterns. For example, it has been mentioned that the disks formed from Gly solutions have a significantly larger count and size than those formed from the LysHCl solutions (recall Fig. 1). It can be both attributed to higher concentration of the used Gly solution and smaller solubility value (see Table 1). More intriguing is the result for Glu solution (Fig. 4) that used exactly the same concentration as LysHCl but showed a qualitatively different network-like pattern corresponding to the simulations with a much higher 2.5-3% global particle concentration (Fig. 5) than 0.5% used to simulate dot-like pattern (Fig. 2). The possible explanation might be in a much lower solubility of the Glu solution (see Table 1). Very low solubility might cause an earlier supersaturation of the drying solvent in the solution, leading to the crystallization of the higher volume of particles in the residual thin film. Full drying of this thin film then leads to the emergence of the network-like structures that have a higher surface coverage than the corresponding dotted patterns.

## Methods

### Experimental techniques

L-Lysine monohydrochloride (LysHCl, 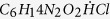) was dissolved in water (Milli-Q) to prepare the aqueous solutions with a concentration of 5 mM. The aqueous solutions of glutamic acid (Glu, *C*_5_*H*_9_*NO*_4_) and glycine (Gly, *NH*_2_*CH*_2_*COOH*) with the same concentration were also prepared in the same way. But the materials have various properties for the deposited amino acids, listed in Table 1^30^,^31^.

The substrate used to deposit the amino acid is atomically flat and hydrophilic mica. Before use, the mica was cut to a square with a size of 1 cm by 1 cm, and was cleaved by using a pair of sharp tweezers. A drop of 2 *μ*l solution was deposited on a flat clean Parafilm and the freshly cleaved mica was placed facing down on the drop. The liquid formed a thin layer of film immediately between the Parafilm and mica, due to its high wettability on mica. After 5 min contact for the adsorption, the mica surface was taken off and dried by a gentle stream of air. The thin liquid layer evaporated, and the mica was imaged by tapping mode AFM (Multimode III, Bruker) in air. The normal spring constant of the cantilever (TESP, Bruker) was 42 N/m, and the nominal radius of the tip is 8 nm. The resonant frequency is around 320 kHz in air. For each sample, multiple areas of 5 *μ*m by 5 *μ*m were scanned by a resolution of 256 × 256 data points. The AFM images were analysed by the offline software to obtain the height and lateral size of the domains.

### Model

**Figure 6:**
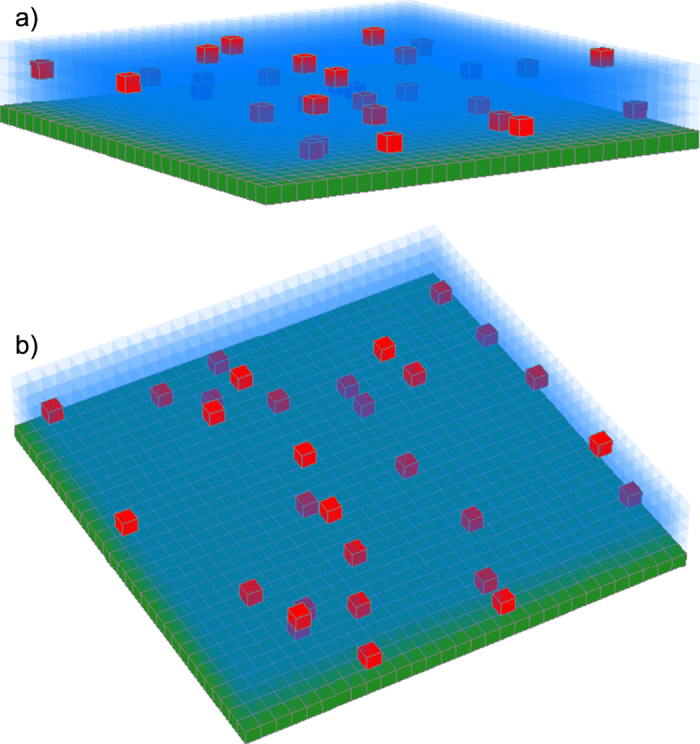
Schematic representation of the coarse-grained simulation domain. The initial state shown in (**a**) isometric view, and (**b**) side view, mimics the thin film of a solution on the substrate.

Three-dimensional (3D) coarse-grained Kinetic Monte Carlo (KMC) model^23^,^26^,^27^ is used to study particle structures formed from the naturally drying solutions. The model in the 3D form is adopted from the initial work of Sztrum *et al.*^27^ with the additional modifications which develop the ideas of Pauliac-Vaujour *et al.*^24^ and Stannard *et al.*^28^. The simulations are performed on a cubical lattice, and each unit cell may be occupied by liquid, vapor, substrate or a particle. The simulation domain is a prism with the square base. The base of the prism has a side length of *D*, and the side wall aspect ratio is *z*. In the domain, the first, lower layer of the cubical lattice is occupied by substrate. In the initial system state, all the cells above the substrate level are filled with liquid or identical cubical particles (see Fig. 6). *l*_*i*_ is the binary variable indicating the presence of solvent at cell number *i*. We assume that *l*_*i*_ = 0 if the cell is occupied by vapor or particle, and *l*_*i*_ = 1 if the cell is occupied by liquid. The corresponding variables indicating the presence of particles or substrate are *n*_*i*_ and *s*_*i*_ respectively. The volumetric concentration of particles is defined by the variable *ϕ*, and the size of each particle is one unit cell. The domain is horizontally periodical, and the side boundaries have toroidal conditions.

The total system energy is given by the Hamiltonian 26: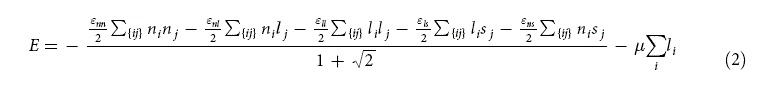 where 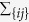 means a sum taken over nearest and next-nearest neighbor cells; *μ* is the system chemical potential; and adjacent liquid cells compose the energy of interaction *ε*_*ll*_, the energy values of interaction between the adjacent liquid-particle and particle-particle cells are *ε*_*ln*_ and *ε*_*nn*_ correspondingly, and *ε*_*ls*_ and *ε*_*ns*_ stand for the liquid-substrate and particle-substrate interactions. Thus, *ε*-values contribute to the total system energy for each corresponding pair of adjacent cells, but for the next-nearest cells the values are scaled by the distance factor, and therefore divided by 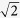 as compared to the nearest cells. There are 6 nearest and 12 next-nearest adjacent lattice cells in the 3D model. The next-nearest cells are considered following the discussion of Sztrum *et al.*^27^, but the next-nearest cells are also counted in the terms including the substrate cells in the present work. Substrate-substrate interactions are excluded from the calculations since they do not change during the entire simulation run. The conditions near the substrate layer are assumed to be similar to the liquid bulk: *ε*_*ns*_ = *ε*_*nl*_, *ε*_*ls*_ = *ε*_*ll*_. The scaling factor in the first term of the right-hand side of Eq. 2 is introduced to keep consistency in *μ* values with the initial model which considered only the nearest cells^27^. The chemical potential, *μ*, is usually set within a range of values where the equilibrium state is vapor. In the described settings it corresponds to the range *μ* < − 3. The dynamics of the system is modeled using the Metropolis algorithm. Each step of the simulation consists of two attempts to make possible moves in the system:

1. Evaporation (condensation) of each liquid (vapor) cell.
2. Random walk of particles within the liquid.

The number of attempts for each particle to make a move in the randomly chosen direction during one Monte Carlo step is defined by the *N*_*mov*_ parameter. The resulting system energy change, Δ*E*, caused by each possible move is calculated using the Hamiltonian expression from Eq. 2, and afterwards the Metropolis probability value is derived from the equation,
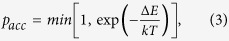
where *T* is the temperature, *k* is the Boltzmann constant and *p*_*acc*_ is the probability that the considered move is accepted in the simulation. If the move is rejected, the system state does not change, and the next possible move is considered. The simulation runs are performed for a variety of parameter values of *μ*, *kT*, *N*_*mov*_ and *ϕ*. The resulted particle structures show the dependence on each of the studied parameters.

## Additional Information

**How to cite this article**: Zhang, X. *et al.* Three-dimensional patterns from the thin-film drying of amino acid solutions. *Sci. Rep.* **5**, 10926; doi: 10.1038/srep10926 (2015).

## Figures and Tables

**Table 1:** Properties of three amino acids deposited from thin-film drying^30^,^31^.

**Amino acid**	**Molar mass**	**Solubility (g/l in water)**	**Diffusion coefficient in water ( × 10^10^ m^2^ s^− 1^)**
Gly	75	249.9 (25 ^*°*^C)	10.554 (0.599 wt % in water)
Glu	147	8.64 (25 ^*°*^C)	6.73 (0.780 wt % in water)
LysHCl	182.65	650 (20 ^*°*^C)	—
